# Small Bowel Stromal Tumors: Different Clinicopathologic and Computed Tomography Features in Various Anatomic Sites

**DOI:** 10.1371/journal.pone.0144277

**Published:** 2015-12-08

**Authors:** Gu-sheng Xing, Shuang Wang, Yue-Min Sun, Zheng Yuan, Xin-Ming Zhao, Chun-wu Zhou

**Affiliations:** 1 Department of Diagnostic Imaging, Cancer Hospital & Institute, Peking Union Medical College and Chinese Academy of Medical Sciences, Beijing, China; 2 Department of Abdominal Surgical Oncology, Cancer Hospital & Institute, Peking Union Medical College and Chinese Academy of Medical Sciences, Beijing, China; 3 Department of Pathology, Cancer Hospital & Institute, Peking Union Medical College and Chinese Academy of Medical Sciences, Beijing, China; University Hospital Llandough, UNITED KINGDOM

## Abstract

Gastrointestinal stromal tumors (GISTs) can present with different clinical and immunohistochemical characteristics according to different anatomic sites. The aim of this study was to compare clinicopathologic and computed tomography (CT) features of small bowel stromal tumors located in the duodenum, jejunum, and ileum. In total, 197 patients (109 male, 88 female) with small bowel GISTs were retrospectively reviewed. All tumors had definite anatomic sites in the small bowel tract with surgical confirmation. The clinicopathologic variables included age, sex, onset of symptoms, and tumor risk category. CT variables included tumor size, degree enhancement, enhancement pattern (region of necrosis), adjacent tissue involvement, lymphadenopathy, and distant metastasis. We assessed any possible differences according to different GIST site of origin. Based on tumor size and mitotic count, the risk categories in different anatomic sites did not differ significantly between duodenal and jejunal GISTs. However, high risk ileum GISTs accounted for 66.0% of ileal cases, which was higher than duodenum cases (36.8%, P = 0.002) and jejunum cases (43.9%, P = 0.004). The mean size of GISTs in the ileum was 9.77 cm, which was significantly larger than in the duodenum (7.41 cm, P = 0.043), and in the jejunum (8.14 cm, P = 0.027). On CT images, enhancement degree appeared to gradually increase from the duodenum to the ileum in the portal phase, and the enhancement pattern presented a tendency for heterogeneity. In Conclusions, the clinicopathologic and CT features of small bowel GISTs can differ according to different primary anatomic sites.

## Introduction

Gastrointestinal stromal tumors (GISTs) are currently the most commonly diagnosed mesenchymal tumor originating from the small bowel tract and its incidence is increasing [1, 2]. GISTs are thought to derive from a precursor of the interstitial cells of Cajal, which express a transmembrane receptor tyrosine kinase encoded by the *KIT* gene, and almost all GISTs express activating mutations in *KIT* that promote tumor survival and growth [2]. CD117 is the protein product of the proto-oncogene c-*kit* [3, 4]. Without immunohistochemical confirmation, most GISTs are misdiagnosed as leiomyomas or leiomyosarcomas.

The diagnosis of small bowel tumors is an ongoing challenge because of a series of disadvantages, such as low incidence, atypical clinical symptoms, a wide spectrum of radiological appearances, polymorphous intestine, and intestine loops overlaps, and thus can be overlooked clinically. Although the small bowel constitutes a significant portion of the gastrointestinal tract, small bowel stromal tumors account for only 20–42% of GISTs [3, 5–7].

GISTs can present with different clinical and immunohistochemical characteristics according to different anatomic sites [8–11]. In general, gastric tumors have a more favorable prognosis than intestinal tumors with similar characteristics [12]. The clinicopathologic features of duodenal GISTs differ from small bowel GISTs, and the former carries a worse prognosis [13]. Although many diagnostic findings of small bowel stromal tumors have been published, few studies have focused on the differentiated analysis of clinicopathologic and imaging features of small bowel GISTs originating from different anatomic sites, including the duodenum, jejunum, and ileum. The aim of this study was to compare the clinicopathologic and CT features of small bowel stromal tumors and to identify the differences or similarities between tumors occurring in the duodenum, jejunum, and ileum.

## Materials and Methods

### Patients

Ethical approval was given by the medical ethics committee of Caner Hospital of CAMS. Due to the retrospective nature of the study, informed conssent was waived. We retrospectively reviewed all data from primary small bowel GISTs with surgical confirmation in our institute (Cancer Hospital, Chinese Academy of Medical Sciences) between September 2005 and March 2015. In total, 197 cases met the inclusion criteria. All 197 patients had complete clinicopathologic and CT data, and the tumors had definite anatomic sites in the small bowel tract. All hospital records data were de-identified and analyzed anonymously in this study. The patients were divided into three groups according to different anatomic site: 68 (34.5%) GISTs originated from the duodenum, 82 (41.6%) from the jejunum, and 47 (23.9%) from the ileum. Tumors were defined as GISTs based on a combination of histological evaluation and CD117 (KIT) positivity. After pathological review, histologic findings were described in accordance with the 7th edition TNM staging of the American Joint Committee on Cancer (AJCC) [14].

### Clinical and Pathology Data

In this study all patients received tumor excision. The surgical procedures were customized based on tumor size, location, and disease extent. Sections of lymph nodes were excised and margin status was assessed in a standard manner. The anatomic sites of the tumors were finally determined by surgical procedure. Of all cases two patients had multiple primary stromal tumors. One patient with duodenal GIST also had a stomach GIST 2 years previously, but the possibility of metastasis was ruled out by the tumors’ different mitotic activities. The other case was confirmed GIST originating from the jejunum and adjacent mesentery with different mitotic activities in the two lesions, and the final diagnosis was synchronous GISTs. In this case we only analyzed the lesions originating from the jejunum.

All clinicopathologic data were evaluated based on case records, operation notes, and pathologic reports. The clinical variables included age, sex, and onset of symptoms. For the sake of contrast analysis, only main symptoms were analyzed, which included incidental finding, epigastria symptoms, GI bleeding, and abdominal mass [3, 15]. Pathologic variables, including tumor size, mitotic figures per 50 high power fields (HPFs), adjacent tissue invasion, necrosis, and lymphadenopathy, were evaluated. Based on tumor size and mitotic count, risk categories were assigned according to the revised NIH criteria, and all the tumors were classified into four levels to assess patient prognosis after surgical resection [16].

### CT Scans

All patients received a standardized spiral CT scan 3–15 days before operative tumor excision. Bowel cleansing is a precondition for CT examination, which includes a low-residue diet or ample fluids the day before the examination and fasting 12–24 hours before examination. A water-soluble solution of 1% Meglumine Diatrizoate was used as an oral positive intraluminal contrast material for most individuals in our practice. Next, 60 and 30 minutes prior to scanning, a total of 750–1000 mL of the oral agent was administered, followed by an additional 500 mL immediately before the scan. In patients with hyper-susceptibility or low-tolerance, 1000 mL to 1500 mL water was used as a negative intraluminal contrast agent instead. A nonionic iodinated contrast agent of Iopromide 62.3% (100 mL, Uitravist-300; Bayer Schering Pharma, Berlin Germany) was injected intravenously at a rate of 3 mL/s via power injection for CT enhancement. CT scan was performed using a single- or dual-phase technique. The first was done with hepatic venous phase imaging 65 s after initiating intravenous injection. The latter was performed with both an arterial phase scan at 25 s and a hepatic venous phase scan at 65 s in patients suspected of upper-abdominal masses. All examinations were performed on a multidetector-row CT with a 16-detector row spiral CT (GE Lightspeed Ultra, GE Medical systems, USA) or a 64-detector row spiral CT (GE Volume Ultra, GE Medical systems). A collimation of 0.75 mm, reconstruction width of 1.25 mm, and a reconstruction interval of 0.8 mm created almost true isotropic volumetric datasets for multiplanar reconstruction (MPR) imaging. All CT imaging data were stored in the picture archiving and communication system (PACS, CAREstream Medical Ltd, Toronto Canada) for further imaging analysis.

### Image Review

Two radiologists evaluated all CT images blinded to clinicopathologic details. The main imaging parameters included: (i) size of the tumor, (ii) degree of tumor enhancement, (iii) portion of necrosis within the tumor, (iv) adjacent tissue involvement, (v) lymphadenopathy, and (vi) metastasis to the mesentery, liver, or more distant sites. To evaluate tumor enhancement more accurately, a new parameter of region-to-muscle ratio (RTM; [CT value of Hounsfield unit in tumor parenchyma]/[CT value in erector spinae muscles]) was adopted. The portion of necrosis within tumors was estimated in CT images and classified into four levels: none or slight necrosis (less than 25%), moderate necrosis (25–50%), obvious necrosis (50–75%), and severe necrosis (more than 75%). A region of low attenuation was considered necrotic, which included cystic areas and non-enhancing tissue (Hounsfield density of less than 25 units). A nodular soft-tissue lesion larger than 10 mm in the short-axis diameter was defined as regional lymphadenopathy. If the metastatic mass was located in the peritoneal or mesenteric region and not adjacent to the main mass, we accepted a diagnosis of peritoneal or mesenteric metastases [17]. As most GISTs appeared with smooth margins, and it was occasionally very difficult to evaluate the tumor growth pattern (round or lobular) and hardly to concur with each other between the two radiologists. These variables were not analyzed in this study.

### Statistical Analysis

Statistical analyses were performed using the Statistical Package for the Social Sciences version 17.0 (SPSS Inc., Chicago, IL, USA). We assessed any possible differences according to different originating GIST site. Categorical variables were compared using *t*-tests, one-way ANOVA for age and CT enhancement. χ^2^ or Fisher’s exact tests for adjacent tissue involvement, lymphadenopathy, and metastasis. *P*-values < 0.05 were considered statistically significant.

## Results

The clinicopathologic characteristics of small bowel GISTs located in different anatomic sites are listed in Table 1.

**Table 1:** Clinicopathologic characteristics of small bowel GISTs in 197 patients.

Variables	Duodenum (n = 68)	P-value ^a^	Jejunum (n = 82)	P-value ^b^	Ileum(n = 47)	P-value ^c^
**Age (years)**	53.97 ± 11.8	0.511	55.58 ± 11.7	0.801	55.3 ± 9.0	0.421
**Sex (Male:Female)**	31:37	0.412	44:38	0.041	34:13	0.007
**Main presentation (*n*, %)**						
Incidental finding	15 (22.1%)	0.454	23 (28.0%)	>0.99	13 (27.7%)	0.514
Epigastria Symptoms	13 (19.1%)	0.002	36 (43.9%)	0.581	18 (38.3%)	0.032
GI bleeding	17 (25.0%)	0.853	22 (26.8%)	0.077	6 (12.8%)	0.154
Abdominal mass	5 (7.1%)	0.567	9 (11.0%)	0.002	16 (34.0%)	<0.001
**Risk category**						
Very low (n, %)	2 (2.9%)	0.590	1 (1.2%)	>0.99	0	0.140
Low	16 (23.5%)	>0.999	19 (23.2%)	0.101	5 (10.6%)	0.091
Intermediate	25 (36.8%)	0.604	26 (31.7%)	0.419	11 (23.4%)	0.155
High	25 (36.8%)	0.869	36 (43.9%)	0.004	31 (66.0%)	0.002

^a,^ P-value between duodenum and jejunum;

^b,^ P-value between jejunum and ileum;

^c,^ P-value between duodenum and ileum.

The age range of the 197 GIST patients was 17–82 years, with a mean age of 53.97 years old in duodenum, 55.58 years old in jejunum and 55.25 years old in ileum. The mean age appeared no significant differences among the three groups. Of all patients the number of male and female is 109 and 88, respectively.72.3% of ileal GISTs occurred in males, which was significantly different from duodenal GISTs (31:37, *P* = 0.007) and jejunal GISTs (44:38, *P* = 0.041).

The clinical symptoms at the time of presentation varied according to tumor location. Duodenal GISTs most frequently presented with GI bleeding (25%), followed by incidental finding (22.1%), epigastric symptoms (19.1%), and abdominal mass (7.1%). For jejunal GISTs, the cardinal symptom included epigastric symptoms (43.9%), incidental finding (28.0%), GI bleeding (26.8%), and abdominal mass (11.0%). For patients with ileal GISTs, the prevalences of symptoms as following: epigastric symptoms (38.3%), abdominal mass (34.0%), incidental finding (27.70%), and GI bleeding (12.8%). The different of clinical symptoms among the three groups are shown in Table 1. Because some patients had more than one symptom, the total number of symptoms was more than the sum of the patients. Moreover, some symptoms were rare and not analyzed, such as small bowel obstruction, weight loss, fever, and anorexia.

Based on tumor size and mitotic count, the risk categories in different anatomic sites did not significantly differ between duodenal and jejunal GISTs. However, high risk grade ileum GISTs accounted for 66.0% of all ileum GISTs, which was higher than duodenum (36.8%, *P* = 0.002) and jejunum (43.9%, *P* = 0.004).

All patients underwent a standardized CT scan procedure in this study. The CT features of small bowel GISTs located in different anatomic sites are listed in Table 2.

**Table 2:** CT findings of small bowel GISTs in 197 patients.

Variables	Duodenum (n = 68)	P-value ^a^	Jejunum (n = 82)	P-value ^b^	Ileum (n = 47)	P-value ^c^
**Size (mean diameter, cm)**	7.41 ± 4.97	0.313	8.14 ± 3.13	0.027	9.77 ± 4.14	0.043
≤5 cm (n, %)	23 (33.8%)	0.480	23 (28.0%)	0.680	11 (23.4%)	0.222
5–10 cm (n, %)	24 (35.3%)	>0.999	29 (35.4%)	0.437	13 (27.7%)	0.423
≥10 (n, %)	21 (30.9%)	0.493	30 (36.6%)	0.016	23 (48.9%)	0.003
**CT enhancement (mean RTM value)**	1.53±0.31	0.016	1.38±0.37	0.310	1.31±0.21	<0.001
**Necrosis**						
≤25% (n, %)	33 (48.5%)	0.003	20 (24.3%)	0.521	9 (19.1%)	0.002
25–50% (n, %)	16 (23.5%)	0.544	15 (18.3%)	0.268	13 (27.7%)	0.666
50–75% (n, %)	11 (16.2%)	0.117	23 (28.0%)	>0.999	13 (27.7%)	0.164
≥75% (n, %)	8 (11.8%)	0.010	24 (29.3%)	0.688	12 (25.5%)	0.079
**Adjacent tissue involvement**	14 (20.6%)	0.276	11 (13.4%)	0.098	12 (25.6%)	0.651
**Lymphadenopathy**	2 (2.9%)	0.204	0	<0.001	0	0.512
**Metastasis**	11 (16.2%)	0.208	7 (8.5%)	0.058	10 (21.3%)	0.624

^a,^ P-value between duodenum and jejunum;

^b,^ P-value between jejunum and ileum;

^c,^ P-value between duodenum and ileum.

In these 197 cases, the mean GIST size appeared to gradually increase from the duodenum to ileum. The mean size of GISTs in the ileum was 9.77 cm (standard deviation [SD] 4.14, range 2.4–23 cm), which was significantly larger than in the duodenum (7.41 ± 4.97, range 1.4–15 cm, *P* = 0.043), and in the jejunum (8.14 ± 3.13, range 2.7–18 cm, *P* = 0.027). In the group with tumor diameters larger than 10 cm, this tendency was more obvious; there were 48.9% (23/47) lesions larger than 10 cm in the ileum, versus 30.9% in the duodenum (*P* <0.001) and 36.6% in the jejunum (*P* = 0.016). The proportion of GISTs less than 5 cm and 5–10 cm was not significantly different between different anatomic sites.

On CT images, GISTs were enhanced more obviously compared with muscles (the mean RTM value was greater than one in all three groups), but the enhancement degree appeared to gradually decrease from the duodenum to the ileum in the portal phase. Duodenal GISTs enhanced most obviously (RTM, 1.53 ± 0.31), followed by the jejunum (RTM, 1.38 ± 0.37; *P* = 0.016) and ileum (RTM, 1.31 ± 0.21; *P* <0.001).

From the duodenum to the ileum, the texture of GISTs presented with a tendency of being heterogeneous. About 48.5% (33/68) of duodenal GISTs had slight necrosis or appeared with homogeneous density (necrotic area less than 25%), versus 24.3% (20/82) in the jejunum (*P* = 0.003), and 19.9% (9/47) in the ileum (*P* = 0.002), while only 11.8% (8/68) of lesions had severe necrosis (necrotic area more than 75%) in the duodenum, versus 29.3% (24/82) in the jejunum (*P* = 0.010) and 25.5% (12/47) in the ileum (*P* = 0.079).

**Fig 1:**
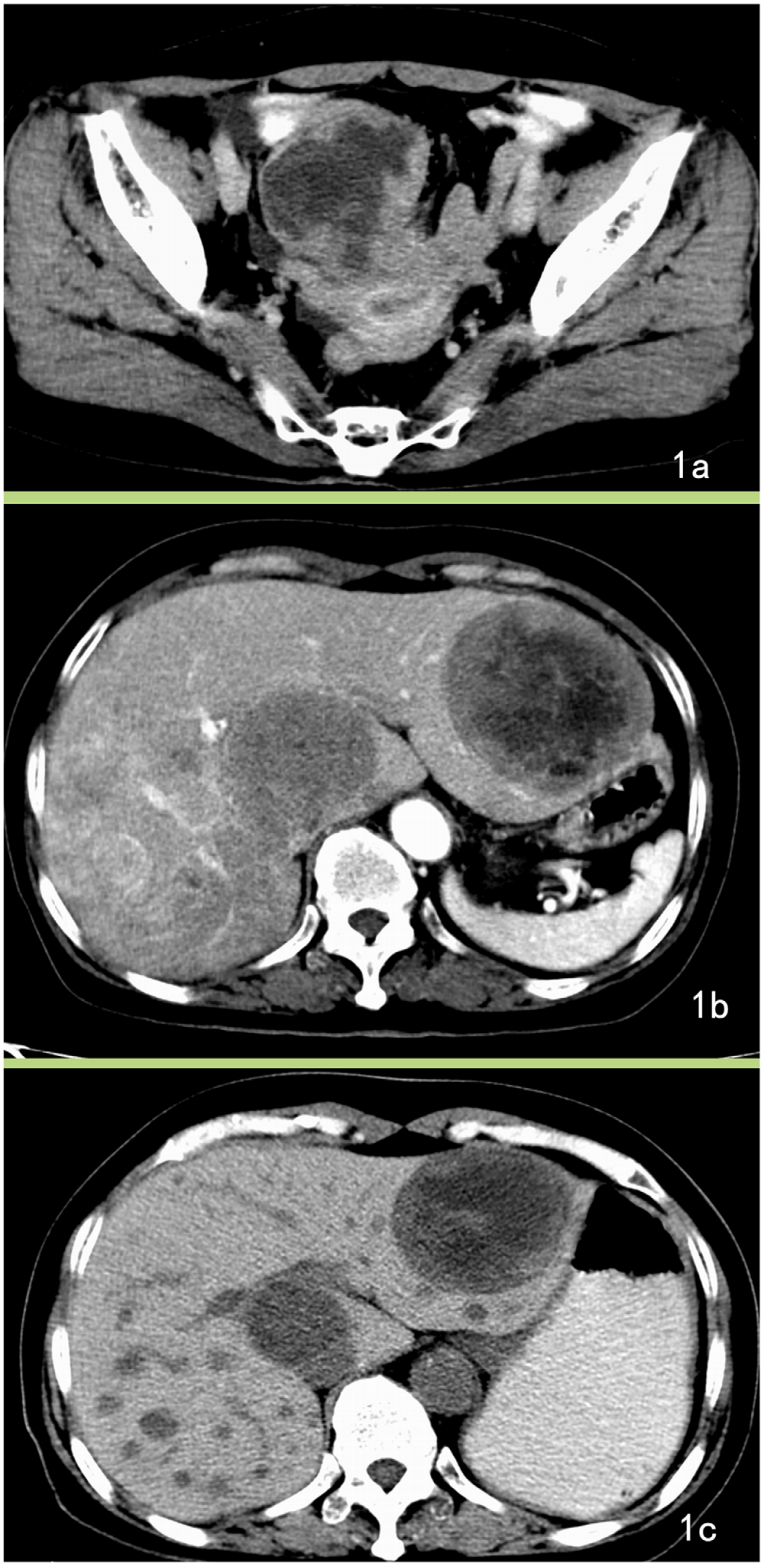
Jejunum GISTs with liver metastasis. A 64-year-old female with jejunum GISTs with liver metastasis presented with clinical abdominal pain for 6 months, and ultrasound examination detected liver masses for 1 week. A. Enhanced CT image reveals a lobular mass with severe necrosis and periphery enhancement. B. The axial section of the liver shows low-density lesions in the liver with slight enhancement. The patient was diagnosed with a malignant stromal tumor and hepatic metastasis. C. After treatment with Gleevec, the neoplasm became cystic and reduced in size.

GISTs can involve adjacent tissue and have the potential of forming distant metastasis and can present with lymphadenopathy, but regional lymph node metastasis is rare. In this series, two patients with duodenal GISTs had peritoneal node enlargement (larger than 10 mm in the short-axis diameter), and demonstrated lymphadenopathy in CT images, but lymph node excision and pathology confirmed these to be steatosis. In total, 28 cases were diagnosed with distant metastases, of which the liver was the most common site (Fig 1); other sites included the peritoneum (n = 12), omentum (n = 7), and lungs (n = 1). Three patient with omental and peritoneal metastasis presented with ascites.

**Fig 2:**
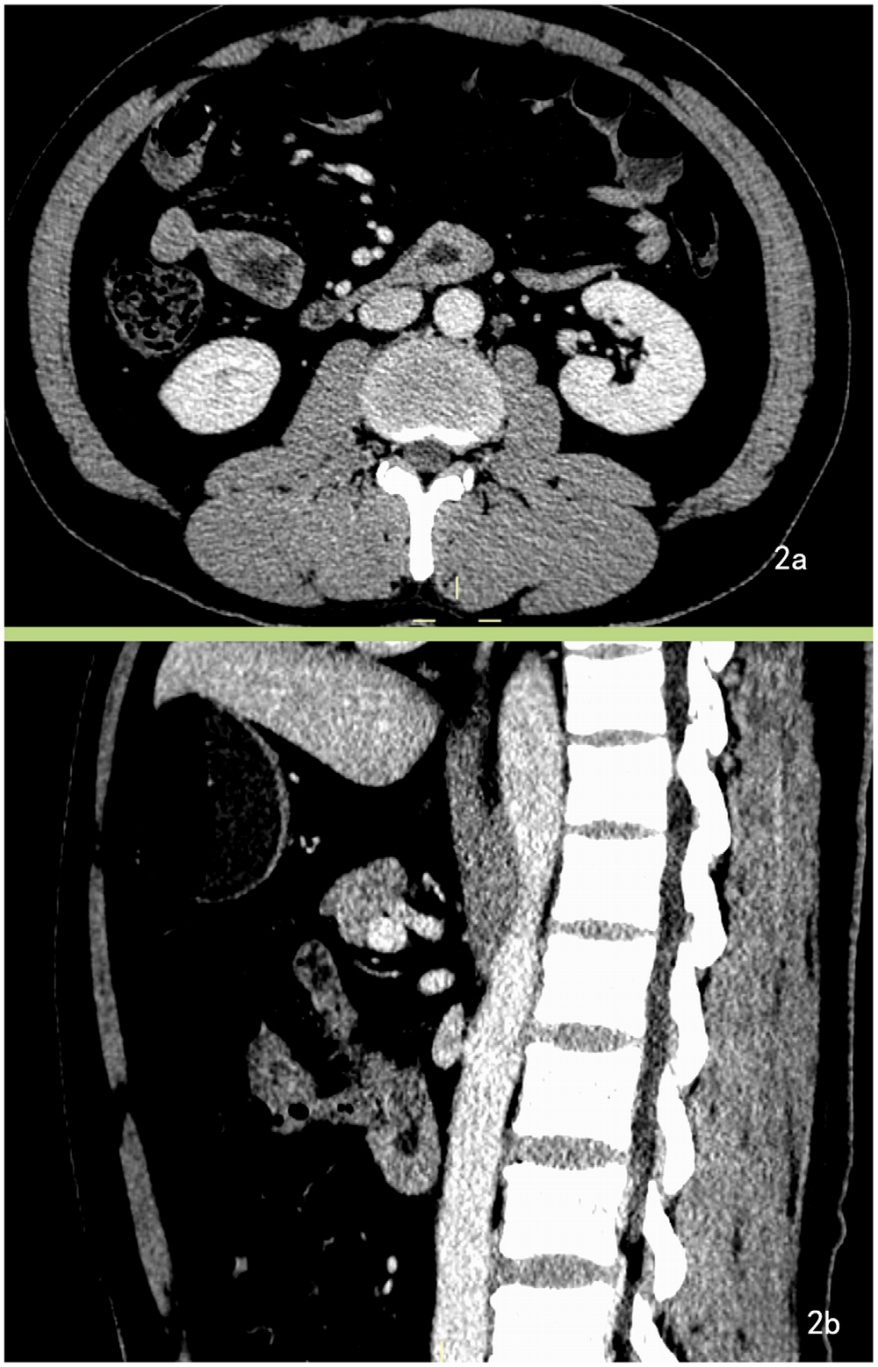
GIST overlooked in CT images. A 64-year-old male with duodenal GISTs presented with melena for the past 20 days. A. Enhanced CT image demonstrates bowel wall thickening at the third segment on the axial section of the CT image and was overlooked. B. Post-procedural CT images (sagittal section) reveal the lesion more clearly. One radiologist had overlooked the lesion, while the other radiologist had misdiagnosed it as a heterotopic pancreas.

One GIST was overlooked in CT images by one radiologist because of its the tiny volume (Fig 2). In some patients, invasion and adhesion was too severe to distinguish the tumor border, and CT post-procedure images help to display the anatomic relationship more clearly. Despite bulky masses in some cases, intestinal obstruction rarely occurred. One case had intestinal obstruction, but the CT did not show corresponding features, such as intestinal dilatation and endo-luminal air-fluid levels.

## Discussion

Small bowl GISTs mainly affect individuals aged from 40 to 70 years [5]. The sex distribution of small bowel stromal tumors is approximately equal. In some studies, males show a slight predominance, but this difference is not significant [3, 18]. In the present series, ileal GISTs tended to occur more often in males compared with other anatomic sites. This result is interesting and requires confirmation with larger sample size.

The onset symptoms of GISTs mostly rely on the size and anatomic location of the tumor [6, 19, 20]. Small bowel stromal tumors grow more slowly than adenocarcinoma, and the clinical symptoms are commonly nonspecific and durative, such as digestive tract bleeding or epigastric symptoms. Duodenal GISTs mostly originate from postbulbar duodenal segments [21], which can be different from duodenal adenocarcinomas as the latter mostly arise from duodenal bulbs and appear as annular lesions at the site of the periampullary [7]. In the present series, about 22.1% (15/68) of duodenal GISTs didn’t have symptoms Compared with duodenal GISTs, jejunum and ileum tumors were more prone to present with epigastric symptoms.

Anemia and periodic melena mostly arise from the ulcerated or necrotic component of tumors. Clinically, a large exoenteric mass may compress the bowel lumen, but seldom induces intestinal obstruction. In this series, only one patient presented with symptoms of an intestinal obstruction, but the CT examination excluded this suspicion.

It has long been controversial whether anatomic site can predict GIST risk category. Some studies have established location as a risk factor for recurrence [9, 10, 22, 23], while others have suggested that duodenal GISTs show earlier presentation, are smaller tumors, and have a lower NIH risk classification than small intestinal GISTs and may have a better prognosis [8]. Miki et al. reported that the prognosis of duodenal GISTs is worse than non-duodenal GISTs [23]. Miettinen et al analyzed 906 GISTs originated from ileal and jejunal and concluded that jejunoileal GISTs seem to have a prognosis similar to duodenal GISTs with comparable size and mitosis parameters[11]. In a recently study, multivariate analysis did not reveal any statistical difference in patients with GIST according to primary location, and patients with duodenal GISTs had a worse prognosis than small intestinal GISTs by univariate analysis [13]. In the present study, we grouped GISTs in more detail according to primary tumor location, and revealed some interesting tendencies with respect to clinicopathologic features. According to the NIH consensus classification system, GISTs located in the ileum are more prone to appear with a higher risk category than tumors in the duodenum and jejunum. However, the risk category of duodenal GISTs did not demonstrate a significant difference compared with jejunum tumors. We speculate that this may be because the duodenum is closer to complicated anatomic structures such as blood vessels, pancreas, stomach, peritoneum, and liver, meaning that the clinical symptoms may be more obvious or appear earlier than in the case of ileal lesions, and may thus be diagnosed at an earlier stage.

Small bowel stromal tumors mainly exhibit an exophytic growth pattern. Combined with the oral administration of an intraluminal contrast agent, CT can objectively demonstrate tumors characteristics, including CT enhancement degree, necrosis, peripheral invasion, and distant metastasis [24]. In most cases, these CT features can differentiated GISTs from other intestinal tumors [3, 25, 26].

Because of mass shrinking after operative excision, CT images usually appear larger than on histological examination. This study only analyzed tumor size measured on CT images. In other reports, the size of GISTs in the duodenum tended to be smaller than those in other sites of the small intestine [13]. In this study, tumor size also demonstrated a tendency of increasing from 7.41 cm in the duodenum and 8.14 cm in the jejunum to 9.77 cm in the ileum. Since large tumor size and high mitotic count are independent adverse prognostic factors [9, 10, 27], this tendency in tumor size suggests a higher risk category for ileal GISTs.

The degree of CT enhancement and enhancement pattern were also associated with primary site in this series. Since increased enhancement on CT scan generally indicates a greater blood supply or more active growth pattern. GISTs located in the duodenum appeared with more obvious enhancement than those in the jejunum and ileum, which may indicate a greater blood supply or a more active growth pattern. However, the enhancement pattern can also be influenced by tumor necrosis, given that CT cannot always differentiate between necrotic and tumor tissue, especially in larger lesions that are more prone to necrosis. Tumors in the duodenum were prone to be homogeneous, while in the ileum they were more prone to be heterogeneous. It suggests that CT enhancement may not be a reliable predictor of risk category or prognosis.

Except for mitotic count and tumor size, differentiating between benign and malignant tumors also depends on neoplasm biological behavior [7], such as adjacent structure invasion and distant metastasis. GISTs are prone to appear with central liquefactive necrosis, which shows as a heterogeneous feature on CT images. Tumor necrosis can also correlate with aggressive behavior [28]. In this series, necrosis was more prone in GISTs located in ileum.

Hepatic metastases can appear with variable appearances on CT images. In the present report, the typical metastases presented with obvious contrast enhancement. Some lesions appeared as cyst-like lesions and might be mistaken for hepatic cysts, but the margins of the metastases were not as distinct and appeared with slight enhancement, and most of the lesions underwent an obvious response to Gleevec treatment. Local lymph metastasis is very rare in patients with GISTs [3, 7]. There were two cases in this series with peritoneal lymphadenopathy on CT images, but the final pathologic examination confirmed that they were steatosis. No patient had confirmed local lymph nodule metastases in this study, which is consistent with previous studies [3, 5].

This study had some limitations. Firstly, all patients in this series underwent enhanced CT scan only, and was lack of non-enhanced CT imaging. Thus, CT signs of hemorrhage and calcification can vary considerably with this method, and thus we did not analyze these indicators. secondly, it was occasionally very difficult to evaluate the tumor growth pattern (round or lobular) and there were disagreements between the two radiologists. As most GISTs appeared with smooth margins, these variables were not analyzed in this study.
